# Risk of Stroke, Dementia, and Cognitive Decline with Coronary and Arterial Calcification

**DOI:** 10.3390/jcm13144263

**Published:** 2024-07-22

**Authors:** Kyari Sumayin Ngamdu, Dinesh K. Kalra

**Affiliations:** Division of Cardiology, University of Louisville School of Medicine, 201 Abraham Flexner Way, Suite 600, Louisville, KY 40202, USA

**Keywords:** atherosclerosis, stroke, cognitive impairment, dementia, computed tomography

## Abstract

Extant research shows that following a cerebrovascular insult to the brain, patients may develop a wide range of cognitive disorders, spanning from mild cognitive impairment (CI) to advanced dementia. Several studies have shown that atherosclerosis in the carotid, coronary, and breast arteries is associated with an increased risk of stroke, CI, and dementia. In this review, we examine the association of subclinical atherosclerotic calcification detected by computed tomography (CT) in these arterial beds and the risk of stroke, CI, and dementia. A major advantage of CT is that it can accurately quantify vascular calcification in different parts of the vasculature during a single examination. However, the strength of the association between CT findings and CI and stroke varies with the location and severity of the arteries involved. Data are still limited on this topic, highlighting the need for additional investigations to further our understanding of the risk of cognitive impairment in patients with subclinical atherosclerosis. It is equally important to test preventive strategies for managing patients in whom vascular calcifications are identified incidentally in randomized controlled trials to study the effects on outcomes, including incidents of stroke and CI.

## 1. Introduction

Extant research shows that, following a cerebrovascular insult to the brain, patients may develop a wide range of cognitive disorders, spanning from mild cognitive impairment (CI) to advanced dementia [1,2,3]. Hence, when patients present with cognitive deficits, a full medical history with a focus on cardiovascular (CV) risk factors and prior CV events needs to be taken to establish if there is a temporal link with a prior cerebrovascular insult. In some cases, neuroimaging might be required to confirm the extent of damage to the brain, while cognitive dysfunction should be assessed through neuropsychological testing [1,4]. The Framingham Heart Study (FHS) showed that after adjusting for age and sex, the incidence of dementia in patients who have had an episode of ischemic stroke is 2-fold higher than in those who have not had a stroke [5]. Evidence further indicates that older age, lower educational attainment, diabetes mellitus, arterial fibrillation, and recurrent stroke significantly increase this risk [5,6,7]. For example, as established by Lobo et al., 1.6% of older individuals (aged ≥65 years) who had a stroke develop dementia [8]. Leys and colleagues concurred with these assertions, adding that the mortality rate is also higher in this population [9]. These factors should thus be considered when allocating resources due to the considerable burden imposed by the care for patients with dementia after a stroke. In particular, focus should be given to the timely identification of individuals with subclinical atherosclerosis to reduce their risk of cerebrovascular disease and subsequent cognitive dysfunction. As demonstrated by Yeboah et al., given that a large proportion of cardiovascular events occur in patients with an estimated 10-year atherosclerotic cardiovascular disease (ASCVD) risk score < 7.5, these individuals should be reclassified using noninvasive imaging studies to improve their health outcomes and preserve their cognitive function [10].

Atherosclerosis is a systemic disease affecting multiple arterial beds. Accordingly, the extent and severity of arterial calcification is a reliable measure of the total atherosclerosis burden on a specific arterial bed [11,12]. Published data further indicate that the progression of the calcification of the arterial vasculature is associated with an increased risk of adverse CVD events [13,14,15]. For example, while those with a coronary artery calcium (CAC) score of zero are regarded to have a lower risk of coronary artery disease (CAD) and death, these risks progressively increase with the increase in CAC score. Thus, the CAC score strongly and independently predicts CAD, stroke, and all-cause mortality [16,17,18,19]. Likewise, an independent and strong correlation of concomitant atherosclerotic disease burden in different arterial beds has been demonstrated [20,21].

**Figure 1:**
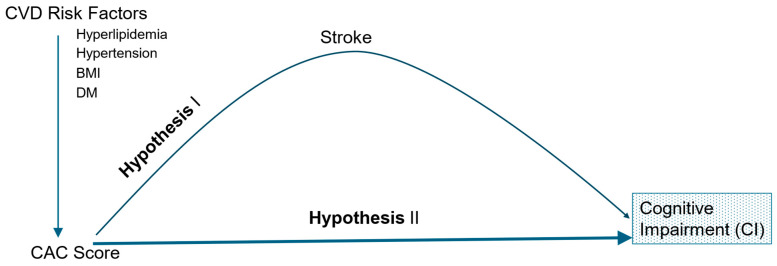
CAC score and cognition. Models for potential association between CAC score and cognitive impairment (CI). Hypothesis I: Potential mediation effects of stroke for the association between CAC score and CI. Hypothesis II: CAC score is independently associated with CI. BMI = body mass index, DM = diabetes mellitus, CAC score = coronary artery calcification score, and CVD = cardiovascular disease.

Several studies also show that atherosclerotic vascular calcification of the coronaries, breast, and carotid arteries, among others, is associated with an increased risk of stroke, CI, and dementia [22,23,24,25]. This causal pathway was examined by the Age, Gene, Environment Susceptibility-Reykjavik Study (AGES-RS) as well as the Cardiovascular Health Study (CHS) [22,23]. Their findings suggest that brain injury strongly mediates the effect of atherosclerosis (based on the CAC score) on CI and dementia (Figure 1), whereas after adjusting for brain injury, this association lessens. These assertions concur with the results obtained in other studies, leading to the conclusion that cerebrovascular insult is a key risk factor for CI [7,26]. Accordingly, in this work, we focus on the extant research on the association of atherosclerotic burden in various arterial beds with stroke, CI, and dementia established noninvasively by computed tomography (CT) scan.

## 2. Methods

To obtain the data needed for this narrative review, we conducted a PubMed database search, focusing on peer-reviewed articles published in the English language in the period spanning from 1 January 1990 to 31 December 2023. The key terms used during the search included [(coronary artery calcification) OR (thoracic aortic calcification) OR (breast arterial calcification) OR (extracranial carotid artery calcification) OR (intracranial carotid artery calcification) AND (stroke) OR (cognitive impairment) OR (dementia)]. Additional articles were also identified from the author’s personal collection and by hand-searching (tracking) the reference lists of the articles produced by the aforementioned search to identify potentially relevant articles. KSN and DKK were responsible for the article selection, full-text review, and risk of bias assessment. The articles that were retained for full review focused on the atherosclerotic vascular calcification burden established using computed tomography angiography (CTA) (of coronary arteries, thoracic aorta, breast arteries, extracranial carotid arteries, or intracranial carotid arteries) as exposure of interest and stroke (self-reported, a medical records review, and/or via brain imaging) or CI/dementia (assessed through cognitive testing, neurological evaluation, medical records/clinically diagnosed CI/Dementia, and medications used to treat dementia) as outcomes of interest. Studies that emerged from the initial search were excluded from further analysis if the appropriate combination of specified exposure(s) with at least one of the three outcomes was lacking. We also excluded meta-analyses, systematic literature reviews, commentaries, conference abstracts, studies in which substantial cases of hemorrhagic stroke were combined with ischemic stroke as an outcome of interest (due to the concerns regarding misclassification), studies that reported stroke as a component of composite CVD outcome, studies in which patients with stroke or invasive coronary interventions comprised the greatest share of the examined cohort, and publications in which only unadjusted estimates were reported.

The articles that met the eligibility criteria were systemically reviewed, aiming to retrieve the following information: study design, sample size, participant age, imaging modality, stroke and/or dementia incidence (in prospective studies), and follow-up duration. Where available, we also recorded the estimated hazard ratio (HR), odds ratio (OR), or Z-scores with 95% confidence intervals (CI) and *p*-values yielded by the most adjusted/parsimonious models, reflecting the conclusions derived from the analyses. All studies included in this investigation were observational and focused on adults aged 18 or above. However, they differed significantly in terms of study designs, populations, the number/type of confounders included in the statistical models, and differences in the definition of exposures/outcomes. Hence, due to the significant heterogeneity, we conducted a narrative review whereby KSN performed data extraction, and the retrieved information was subsequently verified by DKK. The authors jointly worked on determining the certainty of the evidence for all the major findings reported in the selected articles in accordance with the grading of recommendations assessment, development, and evaluation (GRADE) methodology.

## 3. Results

### 3.1. Coronary Artery Calcification

In twelve articles pertaining to eight studies, the authors evaluated the association between the CAC score and increased stroke risk, thus meeting the inclusion criteria in this section [22,23,27,28,29,30,31,32,33,34,35,36]. Four of these studies were conducted in the United States (US), three were performed in Europe, and one study was based in Asia, jointly producing 65,019 participants. The patients’ ages ranged from 44 years in the Walter Reed study to 84 years in the Multi-Ethnic Study of Atherosclerosis (MESA) study [28,36,37]. The follow-up duration, stroke event rates, and estimated HR/OR with a 95% CI for all eight studies are shown in Table 1. As can be seen from Table 1, only in three of the examined articles did the authors report that the CAC score was not significantly associated with stroke [28,29,30]. However, each of these studies had some limitations pertaining to either short-term follow-up periods or relatively low stroke event rates. In two articles reporting on the findings from the Walter Reed study, increased CAC scores were found to significantly increase the risk of stroke in young adults [33,36]. The authors of the reviewed articles further noted that the risk of stroke consistently increased in individuals with persistently high CAC scores. Kim et al. and Mehta et al. similarly found that a CAC score threshold ≥ 100 Agatston units (AU) is independently associated with a significant increase in stroke events [32,35]. In four studies, CAC score–age, CAC score–sex, and CAC score–race/ethnicity interactions for stroke were examined [27,29,30,31,35]. Yet, only Hermann et al. reported a significant CAC–age interaction (subjects ≤ 65 versus >65 years of age [HR = 2.21; 95% CI = 1.59–3.06] versus [HR = 1.11; 95% CI = 0.80–1.59]) while Vliegenthart et al. noted a significant interaction between the CAC score and male (OR = 3.5; 95% CI = 1.5–17.8) but not female (OR = 2.5; 95% CI = 0.7–7.8) sex [27,31]. The latter finding was attributed to fewer stroke events in women, resulting in a wide confidence interval. In five articles, the predictive probabilities of the CAC scores beyond traditional CAD risk factors for stroke events were evaluated [29,31,33,35,36]. In the Heinz Nixdorf Recall study, in individuals with low/intermediate Framingham risk scores (FRS), the CAC score was found to improve stroke prediction accuracy beyond that based on the traditional CVD risk factors [31]. Similarly, in the Walter Reed study, the addition of CAC severity to a model comprising hypertension, hyperlipidemia, diabetes mellitus, tobacco use, and age at baseline improved the predictive accuracy for stroke significantly (C statistics = 0.704; 95% CI = 0.683–0.725; *p* < 0.0001) [33]. 

In eight articles related to five studies, the authors reported on the association between the CAC score and the risk of CI/dementia [22,23,25,37,38,39,40,41]. Three of these studies were conducted in the US, and the remaining two were based in Europe, yielding 24,778 participants in total. The age of the study participants ranged from 18 years in the Pittsburgh Epidemiology of Diabetes Complications (EDC) study to 84 years in the MESA study [37,40]. The follow-up duration, dementia incidence, and estimated HR/OR/Z-scores with 95% CI are shown in Table 1. In three of these studies, the baseline CAC score was found not to be significantly associated with dementia [23,25,39]. In three articles from two studies, the authors reported mixed findings for the association between baseline CAC scores and cognitive functioning measures [22,25,38]. In two articles, focus was given to the link between a CAC score progression and the risk of CI/dementia [40,41]. According to Huang et al., who reported on the findings yielded by the MESA study, a CAC progression over 2.5 years was not significantly associated with an increased risk of dementia after adjustment for demographic variables, vascular risk factors, and a baseline CAC score (HR = 1.10; 95% CI = 0.75–2.04; *p* = 0.4) [41]. On the other hand, based on the findings obtained in a smaller study, Guo et al. concluded that a CAC score progression was a stronger risk predictor than the baseline CAC scores for CI in middle-aged adults with type 1 diabetes mellitus after 14 years [40]. It is also worth noting that the reviewed studies demonstrated a positive dose-graded association between the baseline CAC value and the risk of CI/dementia. In two articles in which the CAC score–age, CAC score–sex, CAC score–race/ethnicity, and CAC score–APOE-E4 status interactions were investigated, no significant interactions for the risk of CI/dementia were found [37,40]. In two articles pertaining to two studies, the association between the CAC score and CI/dementia was found to be mediated by the severity of ischemic brain injury determined by neuroimaging [22,23]. Conversely, in MESA, after excluding interim stroke, associations between the CAC score and risk of dementia remained statistically significant (HR = 1.18; 95% CI = 1.03–1.36; *p* = 0.018) [37]. Additionally, in the Rotterdam study, researchers found no change in the effect sizes and significance for the association between the CAC volume and risk of dementia after censoring for stroke [25].

**Table 1:** Coronary artery calcification and risk of stroke, cognitive impairment, and dementia.

Author, PIMD	Sample Size and Design	Mean/Average Age/Range (Years)	Imaging Modality	Follow-Up (Years)	Number of Incident Cases	Outcome	OR/HR/Z-Scores	95% Confidence Interval	*p*-Value
Vliegenthart et al. [27], 11823653	n = 2013 Cross-sectional	70.8 ± 5.5	EBCT			Stroke	3.0	[1.3, 6.8]	0.007
Folsom et al. [28], 18574091	n = 6698 Prospective	45–84	EBCT/MDCT	5.3	59	Stroke	1.1	[0.8, 1.4]	0.71
Vidal et al. [22], 20360538	n = 4250 Cross-sectional	76.0	CCT			Cerebral infarcts	1.36	[1.10, 1.23]	0.002
						Memory	−0.04	[−0.11, 0.03]	0.56
						Speed of processing	−0.09	[−0.15, −0.002]	0.01
						Executive function	−0.02	[−0.09, 0.05]	0.09
						Dementia	2.34	[1.31, 4.19]	0.04
Rosano et at. [23], 15817006	n = 409 Cross-sectional	78.2	EBCT			Cerebral infarcts	2.31	[1.1, 4.9]	Not given
						MCI/dementia	1.65	[0.8, 3.4]	Not given
Elias-Smale et al. [29], 21606087	n = 2153 Prospective	69.6 ± 6.6	MDCT	3.5 (IQR: 2.5–4.3)	52	Stroke/TIA	0.5	[0.2, 1.0]	Not given
Bos et al. [30], 21868705	n = 885 Cross-sectional	66.7 ± 5.5	MDCT			Cerebral infarct	1.28	[0.95, 1.71]	0.1
Bos et al. [38], 22537801	n = 844 Cross-sectional	69.5 ± 6.7	MDCT			Memory	0.00	[−0.05, 0.03]	0.98
						Speed of processing	−0.03	[−0.06, 0.01]	0.19
						Executive function	−0.02	[−0.06, 0.02]	0.27
						Global cognition	−0.01	[−0.04, 0.02]	0.58
						Motor speed	−0.06	[−0.10, −0.01]	0.01
Hermann et al. [31], 23449263	n = 4180 Prospective	59.2 ± 7.7	EBCT	7.9 ± 1.6	92	Stroke	1.52	[1.19, 1.92]	0.001
Bos et al. [25], 25150731	n = 1847 Prospective	69.4 ± 6.7	MDCT	6.0 ± 0.5	90	Global cognition	−0.02	[−0.05, 0.01]	0.13
						MMSE	−0.16	[−0.27, −0.05]	<0.01
						Dementia	1.12	[0.85, 1.48]	0.43
Kim BJ et al. [32], 21157110	n = 312 Cross-sectional	68.7 ± 3.7	CCT			Silent lacunar infarct	5.04	[1.86, 13.63]	<0.01
Handy et al. [39], 26970999	n = 6814 Prospective	62.2 ± 10.2	EBCT or MDCT	9.5 (IQR: 9.7–10.7)	119	Dementia	1.33	[0.81, 2.19]	Not given
Fujiyoshi et al. [37], 28465455	n = 6293 Prospective	45–84	EBT or MDCT	12.2 (IQR: 11.6–12.7)	271	Dementia	1.24	[1.08, 1.41]	0.002
Guo et al. [40], 30471556	n = 148 Prospective cohort of DMT1	37.2 (Range: 18.3–56.1)	EBCT	14.0 ± 3.5	41	Cognitive impairment	1.7	[1.1, 2.9]	0.03
Mitchell et al. [33], 29153576	n = 23,637 Retrospective	50 ± 8.5	EBCT	11.4 (IQR: 8.5–13.8)	772	Cerebral infarcts	1.9	[1.5, 2.5]	<0.0001
Hillerson et al. [34], 32348185	n = 406 Retrospective, case-control. Cohort of AF	61 (IQR: 55–69)	Non-gated chest CT	0.9 (IQR: 0.5–2.9)		Cerebral infarcts	1.47	[1.10, 1.97]	<0.01
Mehta et al. [35], 32806939	n = 7042 Prospective	56.8 ± 12.7	EBCT/MDCT	12.3 (IQR: 10.9–13.3)	241	Cerebral infarcts	1.42	[1.02, 1.98]	0.039
Javaid et al. [36], 34743556	n = 13,397 Prospective	44	EBCT	11.1	225	Stroke	1.73	[1.01, 2.97]	0.047
Huang et al. [41], 35287946	n = 4173 Prospective	64.0 ± 10	CCT (CAC progression)	13.2 (IQR: 11.2–15.3)	350	CASI score	−1.70	[−3.73, 0.32]	0.10
						Dementia	1.10	[0.75, 2.04]	0.40

AF = atrial fibrillation; CASI = cognitive abilities screening instrument; CCT = cardiac computed tomography; DMT1 = diabetes mellitus type 1; EBCT = electron bean computed tomography; HR = hazard ratio; MDCT = multi-detector computed tomography; MCI = mild cognitive impairment; MMSE = mini-mental state exam; OR = odds ratio.

### 3.2. Thoracic Aortic Calcification (TAC)

In six articles pertaining to four studies (two from Europe and two from Asia), the association between TAC and increased stroke risk was evaluated, thus meeting the inclusion criteria for this section of the literature review [29,30,42,43,44,45]. The total sample size for all four studies was 8644 patients, who ranged in age (mean) from 53.0 to 70.0 years. Table 2 provides details on the follow-up duration, stroke events, and estimated HR/OR with a 95% CI for each study. The obtained findings were incongruent, as TAC and cerebrovascular events were found not to be associated in one study, while a significant association—independent of the traditional CVD risk factors—was noted in two studies [29,44,45]. However, even in the latter case, a further adjustment for calcification in other arterial beds rendered this association insignificant, and in one of these studies, descending (but not ascending) TAC was associated with stroke [44,45]. In four articles from two studies, the authors reported that TAC showed a graded association with the risk of stroke while also providing the TAC–age and TAC–sex interactions for stroke [29,30,44,45]. Yet, in only one of these studies, these interactions were significant for the association between TAC and an increased risk of stroke [45]. In two studies, the authors evaluated the predictive probabilities of TAC beyond the commonly studied CVD risk factors (as reflected by FRS) for stroke events [29,45]. They found that expanding the traditional CVD risk factor model to include TAC did not significantly improve its predictive value of incident stroke when applied to the population in the focus of the investigation. Moreover, based on the data provided by Hermann et al., TAC was unsuitable for discriminating incident stroke in participants with high FRS [45]. 

The association between TAC and the risk of CI/dementia was evaluated in three articles reporting on the Rotterdam study (Europe) and one article pertaining to a US-based study, with 6520 participants in total [25,38,46,47]. The mean age of the study participants ranged from 65 to 70 years [25,47]. The follow-up duration, incidence of dementia, and estimated HR/OR/Z-scores with a 95% CI are shown in Table 2. According to the reported findings, TAC was not significantly associated with dementia, while three articles reported a significant association with cognitive impairment [25,38,47]. The reviewed studies demonstrated a positive dose-graded association between TAC exposure and the risk of CI/dementia [25,38].

### 3.3. Breast Arterial Calcification (BAC)

In four papers in which findings of three studies were reported, the authors evaluated the association of BAC and increased stroke risk in women who had undergone mammography as a part of routine breast cancer screening, thus meeting the inclusion criteria for this section [24,48,49,50]. Three of these studies were conducted in Europe and one in the US, with a total sample of 28,130 women ranging in age from 40 to 79 years. The follow-up duration, stroke events, and estimated HR/OR with a 95% CI are shown in Table 3. In three articles, BAC was defined as the presence (BAC-presence [in at least one breast]) or absence (BAC-absence), while in the remaining article, BAC was scored as absent, mild, moderate, or severe [50]. According to the results obtained in the European Perspective Investigation of Cancer-Norfolk (EPIC-Norfolk) study, BAC-presence was not significantly associated with an increased risk of stroke in women aged above 55 not taking hormone replacement therapy [49]. However, the sample size for this study was small (n = 1590), and only 15 women (9.43%) had a stroke, as reflected by the wide confidence interval (OR = 2.02; 95% CI = 0.61–6.69). Conversely, in the Prospect-EPIC Cohort study, an increased BAC severity was significantly associated with an increased risk of stroke (HR = 3.21; 95% CI = 1.51–6.83) when compared to BAC-absence, whereas the effect size was not significant when the BAC-presence group was compared to the BAC-absence group (HR = 1.43; 95 CI = 0.80–2.58) [50].

### 3.4. Extracranial Carotid Artery Calcification (ECAC)

As a part of two cross-sectional studies and one longitudinal prospective, the population-based study from the larger Rotterdam cohort study, the association between extracranial carotid artery calcification (ECAC) and increased stroke risk was evaluated, thus meeting the inclusion criteria for this section [29,30,44]. The total sample size analyzed across the three papers was 5559, and the mean patient age was 69.7 ± 6.8 years. The follow-up duration, stroke events, and estimated HR/OR with a 95% CI are shown in Table 4. In the longitudinal cohort study, Elias-Smale and colleagues examined the data pertaining to 2153 patients and found that, at a 3.5-year follow-up, an increased ECAC severity was associated with a modestly increased risk of stroke, but the effect size did not reach statistical significance (HR = 1.4; 95% CI = 0.6–3.2) [29]. These authors further reported that the ECAC severity did not significantly improve the stroke event risk prediction in the studied population [29]. On the other hand, the authors of the remaining two articles found an association between a significantly elevated risk of stroke and increasing ECAC severity [30,44]. In all three studies, no significant interaction between ECAC and gender was noted in the stroke risk estimates [29,30,44]. 

In two further studies (one longitudinal and one cross-sectional) from the Rotterdam cohort study, the researchers examined the association between ECAC volume and the risk of CI/dementia. Based on the evaluation of 2691 patients (mean age 69.4 ± 6.7), the follow-up duration, incidence of dementia, and estimated HR/OR/Z-scores with a 95% CI are reported in Table 4. The obtained findings for the cross-sectional study (n = 844) indicate that an increased ECAC volume was significantly associated with a more significant cognitive performance deterioration in the domain of information processing speed and motor speed [38]. In the longitudinal study focusing on 1847 community-dwelling adults, a statistically significantly increased risk of dementia was noted in individuals with a larger ECAC volume at the 6-year follow-up (HR = 1.37; 95% CI = 1.05–1.79) [25]. This effect size remained statistically significantly associated with dementia after censoring for stroke (HR = 1.32; 95% CI = 1.02–1.71). 

### 3.5. Intracranial Carotid Artery Calcification (ICAC)

In one cross-sectional study and one longitudinal cohort study conducted within the Rotterdam cohort study, the association between intracranial carotid artery calcification (ICAC) and increased risk of stroke was evaluated, thus meeting the inclusion criteria for this section [30,51]. According to the analysis of 3208 patients (mean age 66.7 ± 5.5 to 69.5 years), the follow-up duration, stroke events, and estimated HR/OR with a 95% CI were determined, as presented in Table 5. In both studies, a significantly elevated risk of stroke was associated with an increasing ICAC volume. In the longitudinal cohort study, which involved 2323 middle-aged and elderly white persons, a statistically significantly increased risk of stroke was noted after a mean follow-up of 6 years (HR = 1.43 per 1 SD increase in ICAC; 95 CI = 1.04–1.96) [51]. This effect size was independent of the CVD risk factors, the presence of calcifications in other arterial vessels, and the ultrasound carotid plaque score. There was no significant interaction between ICAC and gender in the risk estimate for stroke [30].

The association between ICAC and the risk of CI/dementia was also evaluated in the Rotterdam study (Europe) and was reported in two articles while also being the topic of one article from Asia [25,38,52]. The total sample size analyzed across these studies was 3270 patients, with the mean age ranging from 62 to 69.4 ± 6.7. As can be seen from Table 5, presenting the follow-up duration, incidence of dementia, and estimated HR/OR/Z-scores with a 95% CI, an increased ICAC volume was significantly associated with poorer cognitive scores in the domains of motor speed and global cognition, as well as a lower MMSE score [25,38]. The researchers in the Rotterdam study also found that an increased ICAC severity was associated with a modestly increased risk of dementia after 6.1 years of follow-up, but the effect size did not reach statistical significance (HR = 1.32; 95% CI = 0.98–1.77) [25]. In addition, the findings yielded by the cross-sectional study indicated that a higher intracranial carotid artery Agatston score was significantly associated with CI (OR = 1.06; 95% CI = 1.00–1.13) [52]. 

## 4. Discussion

Based on the studies included in this review of arterial calcification in major vessel beds established via CT as a measure of atherosclerotic burden, the evidence for a significant association with stroke and cognitive impairment (CI) is plausible. A major advantage of CT is that it can accurately quantify vascular calcification in different vessel beds during a single examination. However, the strength of the association between the CT findings and CI and stroke varies with the location and severity of the arterial vessel involved. For example, the association between ECAC and ICAC with stroke remained significant after adjusting for conventional CVD risk factors, the presence of calcifications in other arterial vessels, and the ultrasound carotid plaque score [30,51]. Additionally, the association of arterial calcification in these two vessels with dementia was independent of stroke [25]. Coronary artery calcification (CAC) scores ≥ 100 AU were indicative of a high stroke risk [32,35]. However, the current evidence is insufficient for establishing whether the association between the CAC score and dementia is mediated by stroke [22,23,25,37]. Additionally, in two studies, the CAC score was found to be a superior stroke predictor relative to the established ASCVD risk factors [31,33]. In a meta-analysis of pooled data from the FHS, MESA, and CHS pertaining to 4778 individuals without known coronary heart disease, stroke, and heart failure at baseline (mean age 70.1 years), the CAC score performance was compared to age for incident stroke risk prediction. After 11 years of follow-up, the CAC score modestly improved the prediction of incident stroke; however, the effect size was not statistically significant [53]. Nonetheless, as atherosclerosis is a systemic disease affecting different arterial vessels concomitantly, it is reasonable to presume a synergistic effect of subclinical atherosclerosis in different arterial vessels on the etiology of stroke and post-stroke cognitive impairment. 

The association of carotid artery calcification with CI/dementia, independent of stroke, is supported by the findings obtained in the experimental study conducted by Muhire et al. using a mouse model of atherosclerosis [54]. These authors induced carotid artery calcification by direct application of periarterial calcium chloride without increasing the blood pressure. In the mouse model, carotid artery calcification led to increased arterial stiffness, thus reducing the resting cerebral blood flow, compromising cerebral autoregulation, and increasing the blood–brain barrier permeability in the hippocampus. These adverse changes manifested through cognitive impairment without evidence of cerebral microhemorrhage. In light of these findings, the authors called for further investigations to establish when individuals with arterial stiffness (i.e., subclinical atherosclerosis) should qualify for treatment and to determine the most optimal strategies for protecting the heart and brain during aging. In line with this, the 2018 guidelines for the management of blood cholesterol from the American College of Cardiology/American Heart Association suggest that adults aged 40–75 years without diabetes mellitus and with LDL-C levels ≥ 70–189 mg/dL who are deemed at intermediate risk of 10-year ASCVD should be considered for CAC testing if the results will impact their management [55]. Having such a reliable measure of atherosclerotic burden would facilitate the identification of patients who will benefit from preventive intervention. Therefore, attention to vascular calcium found incidentally during imaging for other purposes in asymptomatic individuals with a significant risk of atherosclerosis might be valuable in the early diagnosis. This might be a potential window of opportunity for shared decision-making with patients regarding aggressive preventive treatment of atherosclerosis, which could possibly delay or prevent full-blown CI/dementia and stroke.

These assertions, as well as the fact that prior reviews were restricted to single arterial beds, have prompted the current narrative review. Our aim was to identify studies examining the association of subclinical atherosclerosis established via CT and the risk of stroke, CI, and dementia in major arterial beds. Due to its comprehensive nature, this review provides a much-needed insight into the complexity of the relationship between subclinical atherosclerosis and the cerebrovascular system. As we employed stringent inclusion criteria, most of the studies included in this review involved community-based cohorts, while those that could potentially lead to selection bias or misclassification were excluded (i.e., investigations in which hemorrhagic stroke was combined with ischemic stroke as the outcome of interest, articles in which stroke was considered a component of composite CVD outcome, studies in which patient cohorts primarily comprised individuals with stroke or invasive coronary interventions, and publications in which only unadjusted estimates were reported). When these restrictions were applied to the identified literature sources, only a few were retained for further review. Moreover, as methods employed to quantify vascular calcification, follow-up duration, and the degree of adjustment for confounding factors in the statistical models differed considerably, such a significant heterogeneity precluded meta-analysis. Accordingly, a narrative review was the most optimal strategy. When interpreting the reported findings, these factors need to be taken into account. Likewise, as most of the included studies were conducted in either Europe or the US, the generalizability of the reported results to other countries with different demographics and risk factors is likely limited. This review also highlights some limitations pertaining to the extant research on this topic, highlighting the need for additional investigations to further our understanding of the risk of cognitive impairment in patients with subclinical atherosclerosis. Future studies should also investigate the predictive probabilities of vascular calcification beyond the traditional CAD risk factors for stroke events and include the use of tools that allow for the automated quantification of atherosclerotic burden and its composition to provide a targeted therapeutic strategy to patients at high risk of dementia due to vascular disease. As the currently available Framingham risk model was not developed for stroke risk prediction, the obtained results could inform the development of more efficient tools and targeted strategies for the prevention of cognitive impairment in patients with subclinical atherosclerosis. In this regard, the pooled cohort equations and the PREVENT calculator are more suited to predict the risk of not only cardiac events and heart failure but also stroke [56,57].

In conclusion, the observational studies included in this review point to an association between vascular calcification and increased risk of stroke, CI, and dementia. However, as the data are still limited, these links are only tentative in the cases of TAC and BAC, necessitating research into the predictive probabilities of vascular calcification beyond the traditional CAD risk factors for stroke events. It will also be equally important to validate treatment protocols for efficiently managing patients in whom vascular calcifications were identified incidentally during imaging for other purposes. Thus, there is a need for randomized trials in this space to evaluate whether preventive treatment (e.g., with statins), once asymptomatic calcification is identified, can prevent dementia, CI, and stroke.

## Figures and Tables

**Table 2:** Thoracic aortic calcification and risk of stroke, cognitive impairment, and dementia.

Author PIMD	Sample Size and Design	Mean/Average/Median/Range Age (Years)	Imaging Modality	Median/Mean Follow-Up (Years)	Number of Incident Cases	Outcome	OR/HR/Z-Scores	95% Confidence Interval	*p*-Value
Itani et al. [42], 16723795	n = 2618 Retrospective	52.9 ± 13.8	Mobile helical CT		28	Stroke	3.31	[1.3, 8.5]	<0.05
Tane et al. [43], 17097748	n = 445 Prospective cohort with hypertension	65.7 ± 5.8	Spiral CT	3.0	27	Stroke	4.9	[1.8, 13.5]	0.001
Elias-Smale et al. [44], 20643406	n = 2518 Cross-sectional	69.7 ± 6.8	MDCT		96	Stoke	1.2	[0.9, 1.8]	Not given
Elias-Smale et al. [29], 21606087	n = 2153 Prospective	69.6 ± 6.6	MDCT	3.5 (IQR: 2.5–4.3)	52	Stroke/TIA	0.7	[0.3, 1.7]	Not given
Bos et al. [30], 21868705	n = 885 Cross-sectional	66.7 ± 5.5	MDCT			Cerebral infarct	1.41	[1.02, 1.94]	0.04
Bos et al. [38], 22537801	n = 844 Cross-sectional	69.5 ± 6.7	MDCT			Memory	−0.05	[−0.10, −0.01]	0.02
						Speed of processing	−0.07	[−0.10, −0.03]	<0.001
						Executive function	−0.06	[−0.01, −0.03]	<0.001
						Global cognition	−0.06	[−0.09, −0.03]	<0.001
						Motor speed	−0.06	[−0.10, −0.01]	0.01
Bos et al. [25], 25150731	n = 1847 Prospective	69.4 ± 6.7	MDCT	6.0 ± 0.5	90	Global cognition	−0.01	[−0.04, 0.02]	0.39
						MMSE	−0.11	[−0.22, −0.01]	0.03
						Dementia	1.27	[0.93, 1.73]	0.13
Hermann et al. [45], 25550362	n = 3930 Prospective	45–75	EBCT	9.1 ± 1.9	101	Stroke	1.09	[1.0, 1.19]	0.04
Wolters et al. [46], 27767996	n = 2428 Prospective	69.5	MDCT	9.3 (IQR: 7.9–9.8)	160	Global cognition	0.04	[−0.05, 0.13]	Not given
						Dementia	0.89	[0.63, 1.26]	Not given
H.E Chung et al. [47], 34113789	n = 1401 Retrospective	65 (IQI: 61–68)	LCSCT	5.0	110	Cognitive impairment	1.13	[1.03, 1.23]	0.009

CT = computed tomography; EBCT = electron bean computed tomography; HR = hazard ratio; IQI = interquartile interval; LCSCT = lung cancer screening CT; MDCT = multi-detector computed tomography; MMSE = mini-mental state exam; OR = odds ratio.

**Table 3:** Breast artery calcification and risk of stroke, cognitive impairment, and dementia.

Author, PIMD	Sample Size and Design	Mean Age	Imaging Modality	Follow-Up	Number of Incident Cases	Outcome	OR/HR	95% Confidence Interval	*p*-Value
Van Noord et al. [48], 9061280	n = 12,239 Cross-sectional	50–69	Screening mammograms			Stroke	1.4	[1.1, 1.8]	Not given
Iribarren et al. [24], 15186654	n = 12,761 Prospective	40–79	Screening mammograms	24.8 (1–32)	1388	Stroke	1.41	[1.11, 1.78]	0.004
Kataoka et al. [49], 16794158	n = 1590 Cross-sectional	61.6	Screening mammograms			Stroke	2.02	[0.61, 6.69]	Not given
Hendriks et al. [50], 25720630	n = 1540 Prospective (case-cohort)	50–69	Screening mammograms	13.2 (IQR: 12.2–14.2)	398	Stroke	2.85	[1.32, 6.15]	0.003

HR = hazard ratio; IQI = interquartile interval; OR = odds ratio.

**Table 4:** Extracranial carotid artery calcification and risk of stroke, cognitive impairment, and dementia.

Author, PIMD	Sample Size and Design	Mean Age	Imaging Modality	Follow-Up	Number of Incident Cases	Outcome	OR/HR/Z-Scores	95% Confidence Interval	*p*-Value
Elias-Smale et al. [44], 20643406	n = 2521 Cross-sectional	69.7 ± 6.8	MDCT		96	Stroke	1.7	[1.2, 2.4]	<0.001
Elias-Smale et al. [29], 21606087	n = 2153 Prospective	69.6 ± 6.6	MDCT	3.5 IQR: 2.5–4.3	52	Stroke/TIA	1.4	[0.6, 3.2]	Not given
Bos et al. [30], 21868705	n = 885 Cross-sectional	66.7 ± 5.5	MDCT			Cerebral infarct	1.51	[1.18, 2.02]	<0.01
Bos et al. [38], 22537801	n = 844 Cross-sectional	69.5 ± 6.7	MDCT			Memory	−0.02	[−0.06, 0.03]	0.39
						Speed of processing	−0.04	[−0.08, −0.01]	0.02
						Executive function	−0.03	[−0.06, 0.00]	0.9
						Global cognition	−0.02	[−0.05, 0.01]	0.16
						Motor speed	−0.10	[−0.14, −0.06]	<0.001
Bos et al. [25], 25150731	n = 1847 Prospective	69.4 ± 6.7	MDCT	6.0 ± 0.5	90	Global cognition	−0.02	[−0.05, 0.01]	0.22
						MMSE	−0.07	[−0.17, 0.04]	0.22
						Dementia	1.37	[1.05, 1.79]	0.02

HR = hazard ratio; IQR = interquartile range; MDCT = multi-detector computed tomography; MMSE = mini-mental state exam; OR = odds ratio.

**Table 5:** Intracranial carotid artery calcification and risk of stroke, cognitive impairment, and dementia.

Author, PIMD	Sample Size and Design	Mean Age	Imaging Modality	Follow-Up	Number of Incident Cases	Outcome	OR/HR/Z-Scores	95% Confidence Interval	*p*-Value
Bos et al. [30], 21868705	n = 885 Cross-sectional	66.7 ± 5.5	MDCT			Cerebral infarct	1.63	[1.20, 2.21]	<0.01
Bos et al. [38], 22537801	n = 844 Cross-sectional	69.5 ± 6.7	MDCT			Memory	−0.03	[−0.07, 0.02]	0.24
						Speed of processing	−0.02	[−0.06, 0.01]	0.19
						Executive function	−0.03	[−0.07, 0.00]	0.05
						Global cognition	−0.03	[−0.06, 0.00]	0.05
						Motor speed	−0.06	[−0.10, −0.02]	0.004
Bos et al. [25], 25150731	n = 1847 Prospective	69.4 ± 6.7	MDCT	6.0 ± 0.5	90	Global cognition	−0.03	[−0.06, 0.00]	0.03
						MMSE	−0.12	[−0.22, −0.01]	0.03
						Dementia	1.32	[0.98, 1.77]	0.07
Bos et al. [51], 24535643	n = 2323 Prospective	69.5	MDCT	6.1	91	Stroke	1.43	[1.04, 1.96]	Not given
Kao et al. [52], 26426620	n = 579 Cross-sectional	62.0	MDCT			Cognitive impairment	1.06	[1.00, 1.13]	0.04

HR = hazard ratio; MDCT = multi-detector computed tomography; MMSE = mini-mental state exam; OR = odds ratio.
